# Interferon-Dependent and Respiratory Virus-Specific Interference in Dual Infections of Airway Epithelia

**DOI:** 10.1038/s41598-020-66748-6

**Published:** 2020-06-24

**Authors:** Manel Essaidi-Laziosi, Johan Geiser, Song Huang, Samuel Constant, Laurent Kaiser, Caroline Tapparel

**Affiliations:** 1 Department of Microbiology and Molecular Medicine, Faculty of Medicine, University of Geneva, Geneva, Switzerland; 2 Division of Infectious diseases, Geneva University Hospital, Geneva, Switzerland; 3 Epithelix Sàrl, Plan les Ouates, Geneva, Switzerland

**Keywords:** Viral host response, Viral immune evasion, Virus-host interactions

## Abstract

Many respiratory viruses cocirculate in the population and multiple infections are commonly reported. The clinical impact of coinfection is unclear and may vary depending on the viral couples involved. Using three-dimensional reconstituted human airway epithelia and clinical viral strains, we investigated the interaction between influenza virus (Flu), respiratory syncytial virus (RSV) and rhinovirus (RV). We showed that Flu and RSV interfere with RV replication, whereas RV does not interfere with either of these viruses. We then experimentally demonstrated that, when present, the interference is not related to a block of viral entry but rather to type I and type III interferon (IFN), the front-line antiviral defense of the respiratory mucosa. Consistent with this observation, we highlighted the differential sensitivity of each virus to IFNs, with RV being the only virus significantly inhibited by IFN-λ and the most sensitive to IFN-α. Finally, as type III IFN is of therapeutic interest due to its low proinflammatory profile, we also assessed and confirmed an inhibitory effect of IFN-λ in the context of persistent RV infections. The present work provides mechanistic clues concerning innate immunity involvement during respiratory virus interactions and confirms that IFN-λ is a promising candidate in the treatment of RV infections.

## Introduction

Viral respiratory infections constitute an important health concern worldwide. Many respiratory viruses cocirculate in the population, and coinfections are commonly reported. However, the type(s) of interaction between distinct viruses, the mechanism and consequences of these multiple infections on disease severity have not been clearly established yet.

Adaptive immunity is not expected to impact coinfections by distantly related viruses while competition for cellular processes (such as nucleotide and lipid biosynthesis, carbon metabolism, protein synthesis…), and/or innate immunity may play a role. Numerous epidemiological studies have described either positive or negative associations between given respiratory viruses based on statistical analyses, with some pairs of viruses being frequently co-detected in patients and others very rarely^1–8^. In line with these observations, experimental coinfections have also put forth different types of viral interactions, from unidirectional of mutualistic inhibition of growth due to competition for cellular resources^9^ to synergism^10^. The role of innate immunity in viral interference has also been illustrated both in old studies^11,12^ and in recent mathematical simulations^13^. However, the actual impact of the antiviral action of interferons (IFNs) and in particular of type III IFN in respiratory virus-specific interference has not been studied in depth in a relevant model.

The human airway epithelium is the first target and also the first barrier to respiratory infections. Mucociliary clearance constitutes a mechanical obstacle to invasion, while the response of infected epithelial cells serves as a second line of defense. Viral detection by the pattern recognition receptors (PRRs) induces the rapid production of type I (α and β) and type III (λ) IFNs^14,15^. Following the binding of their receptors, type I and type III IFNs trigger a common signaling pathway that leads to the expression of IFN-stimulated genes and the establishment of an antiviral state. Recent studies have emphasized the key role of IFN-λ in the antiviral defense properties of the respiratory and intestinal mucosae^16–18^.

Rhinovirus (RV), influenza virus (Flu), and respiratory syncytial virus (RSV) are the most frequent etiological agents of acute respiratory infections^19^. In agreement with their high prevalence, RV, Flu and RSV are frequently detected in multi-infection cases. The mechanisms and consequences of these coinfections for the host require further investigation. Recently, we compared the pathogenesis of extremely common respiratory viruses in *in vitro* reconstituted human airway epithelia and highlighted virus-specific infection signatures^20^. We observed that Flu induced ciliated cell loss, tissue integrity disruption and was a strong cytokine inducer while RSV and RV (with the exception of RV from the B species) altered cilia beating and induced intermediate cytokine response. In the present research, we aimed to go a step further and investigate the interactions between Flu or RSV, and RV in the context of co- or sequential infections using the same highly relevant tissue culture model and clinical viral strains isolated directly from infected respiratory samples. We were able to show that RV infection is inhibited by RSV and Flu, while neither of these viruses is affected by pre- or coinfection with RV. Of note, RV infection is unaffected by prior or coinfection with coronavirus OC43, a virus inducing very low tissue response^20^. We then addressed the mechanisms underlying these observed differences and demonstrated a key role of the host’s type I and type III IFN response. In line with this, we showed that, in contrast to RSV and Flu, RV is highly sensitive to IFNs and particularly to type III IFN. As this latter is known to induce less inflammation and side effects than type I IFN in the infected host and could thus be of therapeutic interest^21^, we tested its effect against RV and showed that this cytokine can significantly impair RV replication during viral persistence in respiratory tissues.

## Results

### H1N1 and RSV-A interfere with RV-A16 replication, while RV-A16 does not interfere with either of these viruses

**Figure 1:**
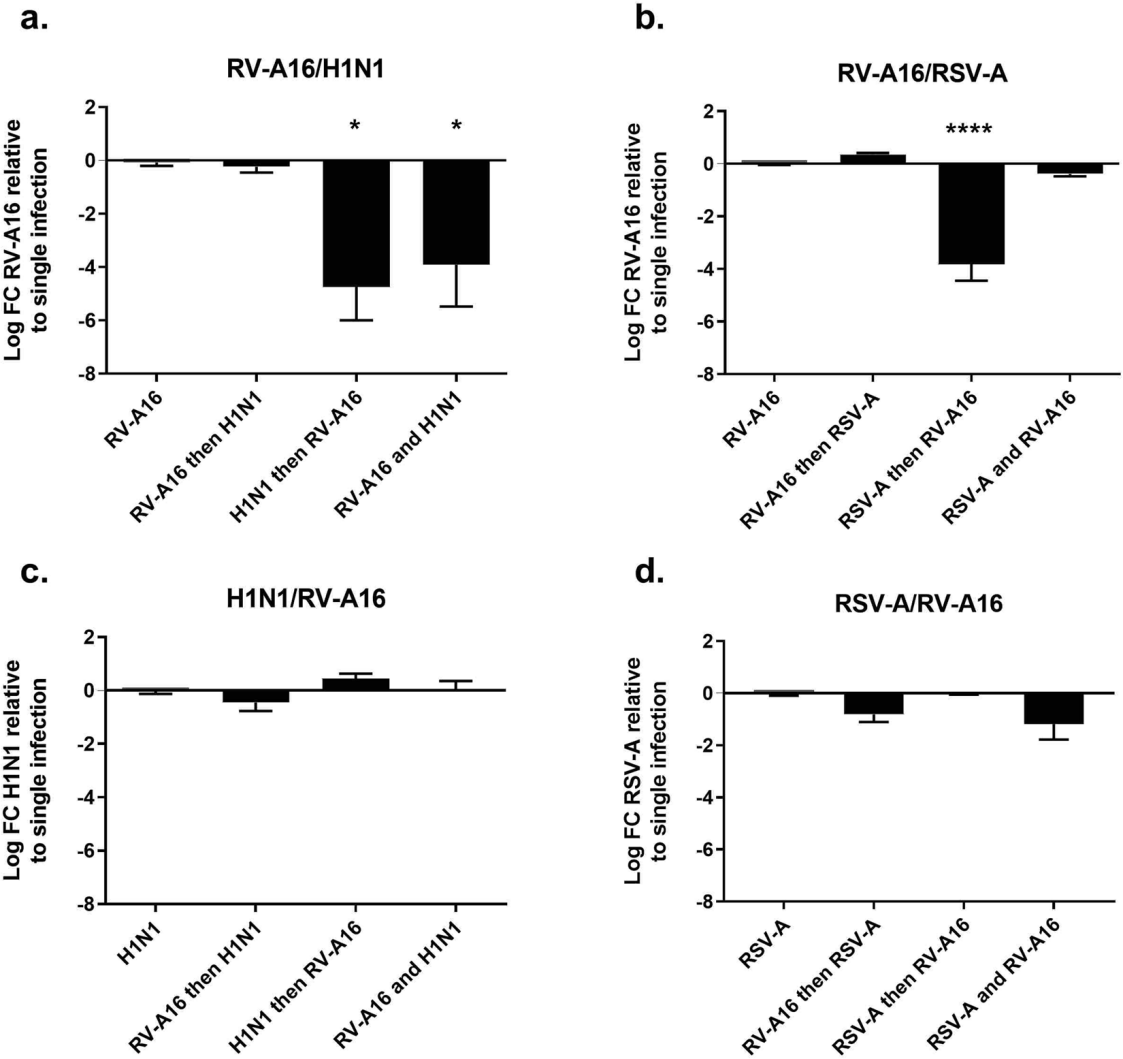
Change in viral replication in dual versus single infections of reconstituted human airway epithelia. Each virus was inoculated alone or in combination, at the same time or two days after the first virus. For each condition, the log fold change (FC) in apically released virus (measured by RT-qPCR five days post infection) in dual versus single infection is indicated on the Y-axis. The analyzed viral couple is specified on the top of each panel while the chronology of infection is shown on the X-axis (‘then’, after two days; ‘and’, at the same time). Statistical significance relative to single infection was calculated using one-way ANOVA (****P < 0.0001, *P < 0.05). Data are expressed as mean and SEM of at least three replicates.

To assess the type of interaction between RV and other respiratory viruses, we tested the replication of RV-A16 in the context of co- or sequential infections with RSV-A and Flu H1N1. Viral stocks were produced in human airway epithelia reconstituted from different healthy donors and the infectious titer of each stock was estimated by endpoint dilution assay in tissues. Co- or sequential infection (with a two-day interval) between RV-A16 and each of the other viruses (using a MOI of around 0.01 viral particles per accessible cell) were performed in reconstituted human airway epithelia and both tissue response (Figs. S1 and S2) and viral replication (Fig. 1) were compared in the presence or absence of the other virus at five days post infection (DPI). The tissue response was neither attenuated nor exacerbated in dual versus single infections. Lactase dehydrogenase (Fig. S1) and cytokine (Fig. S2) release were not different in dual versus single infections. In contrast, we observed differential viral interferences. RV-A16 replication was decreased by respectively 4.7-log and 3.9-log at five DPI if the virus was inoculated two days after or at the same time as H1N1 (Fig. 1a). RV-A16 replication was also affected by prior infection with RSV-A (3.8-log reduction) (Fig. 1b). In contrast, RV-A16 pre- or coinfection did not interfere significantly with the replication of any of the other viruses tested (Fig. 1c,d).

### The observed interference is not related to a block of viral binding or entry

**Figure 2:**
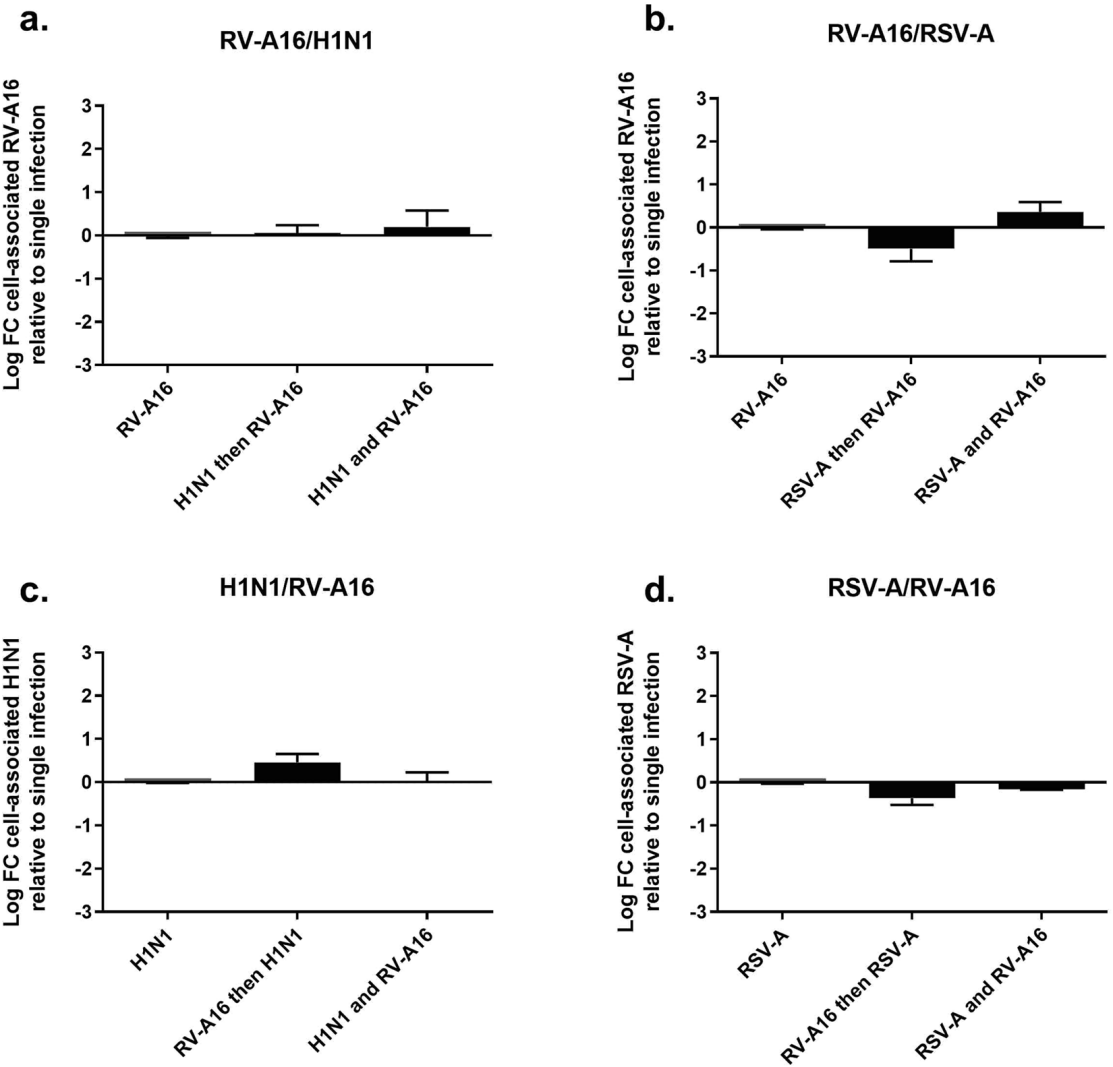
Change in cell-associated virus in dual versus single infections of reconstituted human airway epithelia. Each virus was inoculated alone or in combination, at the same time or two days after the first virus. For each condition, the log fold change (FC) in cell-associated virus (measured by RT-qPCR four hours post inoculation) in dual versus single infection is indicated on the Y-axis. The analyzed viral couple is specified on the top of each panel, while the chronology of infection is shown on the X-axis (‘then’, after two days; ‘and’, at the same time). Statistical significance relative to single infection was calculated using one-way ANOVA. Data are expressed as mean and SEM of at least at least three replicates.

To elucidate whether viral binding or entry can be impaired in the context of co- or sequential infection, tissues were infected for four hours, extensively washed to remove unbound viruses, and lysed to quantify cell-associated viruses. This was done for both simultaneous and sequential infections (Fig. 2). In the latter condition, the cell-associated virus was quantified four hours after inoculation of the second virus, while efficient replication of the first virus was confirmed by RT-qPCR. We did not observe a consistent nor a significant reduction in the number of cell-associated viruses in the context of either co- or sequential infections as compared with single infections (Fig. 2). This suggests that the first infection does not prevent binding and/or entry of the second virus and that another mechanism accounts for the interference observed in Fig. 1.

### Type I and type III IFNs released by infected tissues interfere with RV-A16 replication

**Figure 3:**
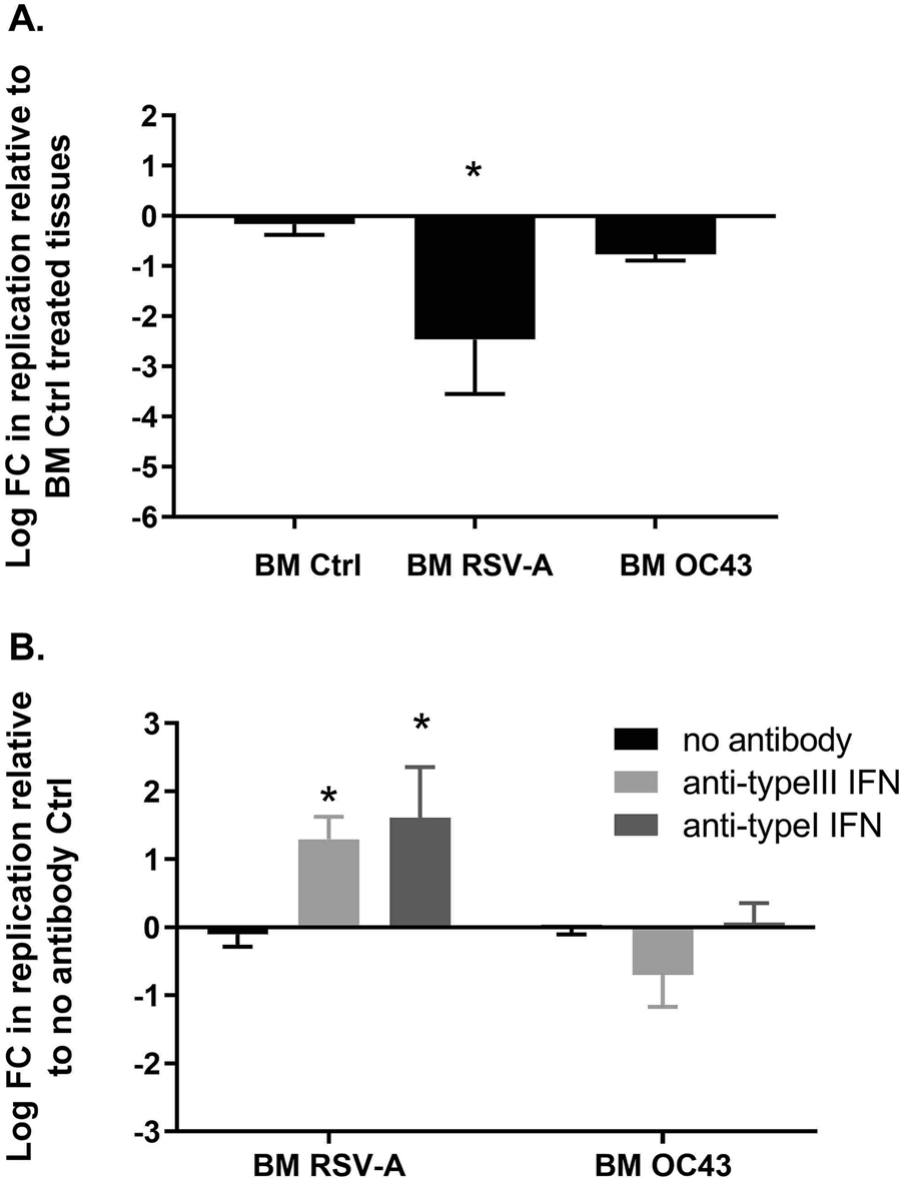
The presence of type I and type III IFNs in RSV-infected medium interferes with RV replication. **A** Basal medium (BM) from tissues infected for two days with RSV-A (BM RSV-A) or OC43 (BM OC43) or from non-infected control (BM Ctrl) were transferred to new tissues for 24 hours before infection with RV-A16. For each condition, the log FC in apically released RV-A16 (measured by RT-qPCR three days post infection) relative to BM Ctrl-treated tissues is indicated on the Y-axis. **B** Same as a but in absence (black bars) or in presence of anti-type I (dark grey bars) or anti-type III (light grey bars) IFN-neutralizing antibodies. For each condition, the log FC in apically released RV-A16 (measured by RT-qPCR three days post infection) relative to BM RSV or BM OC43-treated tissues in the absence of neutralizing antibodies is indicated on the Y-axis. Statistical significance relative to Ctrls was calculated using one-way ANOVA (*P < 0.05). Data are expressed as mean and SEM of two replicates.

To find out whether viral interference could be attributed to the synthesis of inhibitory molecules by infected tissues, we transferred the basal medium of tissues infected for two days with RSV-A or of non-infected controls to new batches of tissues. After a one-day incubation period, the tissues were apically infected with RV-A16. The replication of RV-A16 was analyzed at three DPI in each condition. As an additional control we included basal medium from tissues infected with OC43. Indeed, as previously published^20^ and as confirmed here (Fig. S3), this virus is a poor cytokine inducer and does not interfere with RV replication. The absence of RSV-A or OC43 in apical washes was confirmed by RT-qPCR to exclude viral carryover from the transferred basal samples. Similar experiments could not be performed with H1N1 infected basal medium as viral carryover was highlighted for this virus. A 2.5-log and a 0.8-log reduction in RV replication was observed upon tissue pretreatment with medium collected from RSV- and OC43-infected tissues, respectively (Fig. 3A). Although the assay did not fully recapitulate the sequential infection conditions, as neither the ongoing replication of the first virus nor the infected cells were present during RV replication, it revealed that antiviral molecules released from RSV-infected tissues interfere with the subsequent replication of RV-A16.

Given that IFNs are the first line of defense against viruses in respiratory tissues, we hypothesized that they may play a key role in the differential interference observed for RV. We thus repeated the same experiment, albeit with adding anti-type I and anti-type III neutralizing Ab to the transferred basal medium. The addition of anti-type I and anti-type III IFN Ab abolished the inhibition observed upon transfer of medium originating from RSV-infected tissues but not from OC43-infected tissues (Fig. 3B). This suggests that the RSV interference is indeed mediated by IFNs.

### Respiratory viruses present different levels of susceptibility to IFNs

**Figure 4:**
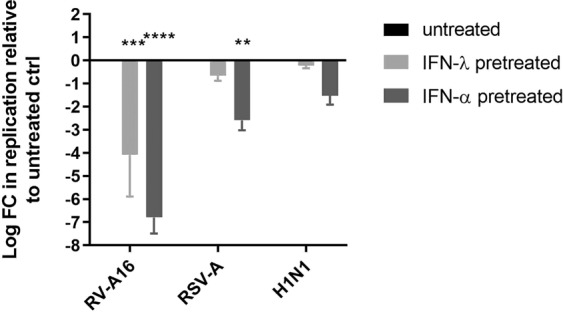
Different susceptibility profiles of respiratory viruses to IFN-λ in reconstituted human airway epithelia. Tissues were treated with IFN-λ or IFN-α before (24 h) and during infection with RV-A16, RSV-A and H1N1. For each condition, the log fold change (FC) in apically released virus (measured by RT-qPCR three days post infection) relative to untreated controls (ctrl) is indicated on the Y-axis. Statistical significance relative to the untreated control was calculated using two-way ANOVA (****P < 0.0001, ***P < 0.001, **P < 0.01). Data are expressed as mean and SEM of at least two replicates.

As RV-A16 replication is inhibited in the presence of other viruses but the reciprocal is not true and as IFNs are involved in this inhibition, we took advantage of the human airway epithelia culture model to assess the differential sensitivity of each of these respiratory viruses to type I and type III IFNs (Fig. 4). Tissues were pretreated with 5 ng/ml of IFN-λ1 [the average concentration measured in basal medium from infected respiratory epithelia^20^] or 2000IU/ml of IFN-α2a for 24 hours and subsequently infected with the different viruses. Fresh IFN was added to the culture medium daily thereafter. As expected, RV-A16 was strongly inhibited by IFN-λ (4-log reduction), while RSV-A and H1N1 were almost not affected (0.7 and 0.2-log reduction respectively) (Fig. 4). A 1 to 2-log higher inhibition was observed for all viruses with IFN-α, but this inhibition remained higher for RV-A16 (6.8-log reduction versus 2.6 and 1.5-log for RSV-A and Flu) (Fig. 4). Decreasing RSV and Flu viral inoculum by 10 and 100-fold respectively (Fig. S4a) or increasing the doses of IFNs by 10-fold (Fig. S4b) did not significantly impact the sensitivity of the viruses to IFN-λ and IFN-α. Of note, different healthy donors were used for these studies which might account for variability in baseline measurements (Fig. S2).

### IFN-λ significantly attenuates RV replication during acute and persistent infection

**Figure 5:**
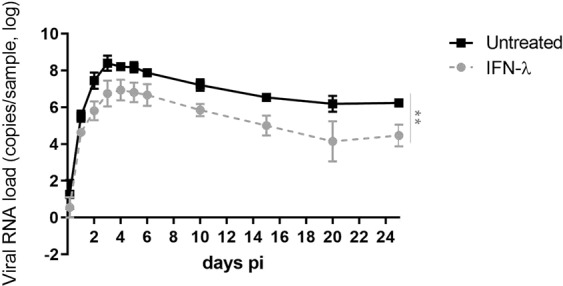
IFN-λ inhibits RV-A16 replication in the context of persistent infections. Infections of airway epithelia were conducted during 25 days in absence or in presence of continuous IFN-λ administration and viral load was quantified using RT-qPCR in apical samples collected at the indicated time points. The statistical significance was calculated with t-tests on the area under the curve (**P < 0.01). Data are expressed as mean and SEM of five replicates.

Our data highlight that, among the tested viruses, RV is the most sensitive to IFN-λ in the presence or absence of the other viruses. RV is known to cause chronic infections in immunocompromised patients and we previously showed that RV infection of respiratory tissues deprived of immune cells also results in RV persistence^20^. As type III IFN induces low inflammation and side effects, it presents a therapeutic interest to treat complicated RV infections. We thus assessed the effects of continuous type III IFN treatment in this persistent infection model. Infections were conducted in presence (with repeated daily administration) or absence of IFN-λ, and RV replication was monitored daily during 25 days. Although less potent than in pretreatment (Fig. 4), cotreatment with IFN-λ significantly reduced RV replication (1- to 2-log) over the whole course of the persistence (Fig. 5).

## Discussion

This study constitutes one of the first deep investigations on the replication and pathogenesis of prevalent respiratory viruses (i.e., RV, RSV and Flu) in the context of dual infections, using a highly relevant *ex vivo* model of reconstituted human airway epithelia and clinical viral strains. This tissue culture model does not contain immune cells but fully recapitulates the first steps of viral respiratory infections. Our results highlight that there is no increase in tissue response in dual versus single infections, but do point to a virus-specific interference.

We showed that RV replication is inhibited by prior infection with RSV-A and H1N1, while RV does not inhibit subsequent infection by these viruses. A number of clinical studies have described viral coinfections, particularly between RV, Flu, and RSV, but the clinical impact remains unclear^22^. Some publications have reported an enhancement in disease severity, while others have suggested the opposite^3,7,8,23–26^. Experimental coinfections have also put forth different viral interactions. Goto and colleagues described a positive association between human parainfluenza virus 2 (HPIV2) and Flu in Vero cells, where HPIV2-induced cell fusion was shown to facilitate Flu spread^10^. On the contrary, coinfections of MDCK cells with RSV and H1N1 highlighted a negative association. Competition for protein synthesis and selective budding from the same infected cells was proposed as the mechanism of the interference^9^. Similarly, RSV and Flu were shown to interfere with each other in a ferret model of coinfection^27^. Competition for cellular resources was also predicted by mathematical modelling of respiratory coinfections^28^. However, competition for cellular resources assumes coinfection of the same cell, which is not frequent in *in vivo* conditions due to the low percentage of infected cells in the respiratory tract^20^.

Our data strongly support the involvement of the antiviral immune response in the observed virus-specific interference. First, virus binding or entry was not affected in sequential versus single infections involving RV, Flu, and RSV (Fig. 2). Second, pretreatment of tissues with basal medium collected from RSV-infected tissues interfered with subsequent RV infection, and this interference was prevented by the addition of anti-type I and -type III IFN-neutralizing antibodies (Fig. 3). Third, RV was the only virus significantly inhibited by IFN-λ and the most sensitive to IFN-α (Fig. 4).

Our observations are in agreement with the observed IFN-λ–mediated inhibition of Sendai virus infection by RSV in differentiated pediatric primary bronchial epithelial cells^29^. In this study, as was seen also in ours, RSV proved much less sensitive to IFN-λ. The authors showed that this was linked with the capacity of RSV nonstructural proteins NS1 and NS2 to block the IFN-λ pathway in a STAT2-dependent manner^29^. Flu and RV also possess interferon antagonists: the Flu NS1 protein interferes with different steps of the IFN signaling cascade^30,31^; and the RV 2A and 3C proteases cleave several antiviral proteins including the PRRs RIG1 and MDA5^32^. Nevertheless, we highlight here, in a unique and highly standardized tissue culture model that faithfully mimics the host respiratory epithelium, that RV is highly susceptible to both pre- and cotreatment with IFNs and is thus less potent than Flu or RSV to counter this host response.

Type I IFN has a more efficient anti-viral effect as pretreatment with this cytokine affected strongly RV and to lower extent RSV and H1N1. To reproduce physiological conditions, the concentration of IFN-λ1 used in pre- and cotreatment were based on the value measured by ELISA in RSV-A-infected airway epithelia^20^. However, increasing the concentration of both type I and type III IFN or decreasing RSV and Flu inocula did not significantly increase their sensibility to these cytokines (Fig. S4). Of note, in this tissue culture model, induction of type I IFN is much less important than that of type III^20^. The positive effect of anti-type I IFN antibodies on RV replication when added to RSV-infected medium was thus unexpected (Fig. 3) and suggests that a slight type I IFN induction is sufficient to interfere with RV infection.

Type I and type III IFNs bind different receptors but trigger a common signaling pathway that leads to the establishment of an antiviral state. Acting in a paracrine fashion, the IFNs induce an antiviral state not only in infected cells but also in cells proximal to the infection, thus controlling both intracellular replication and viral spread^17,18^. Type I IFN targets nearly all immune cells and induces a strong inflammatory response often detrimental for the host. In contrast, due to the limited expression of its receptor, type III IFN only acts at the site of viral replication, the epithelial barrier, and on few innate immune cells, without activating a massive inflammatory response and therefore without compromising host fitness^33^. PEG-IFN-λ passed phase II clinical trials for hepatitis C treatment, displaying an attractive pharmacological profile^34^. This cytokine has been shown to reduce RV replication *in vitro*^35,36^ and in primary bronchial epithelial cells^37^. Our data with a clinical RV strain and 3D *in vitro*–differentiated epithelia confirm that IFN-λ is a promising therapeutic candidate for controlling RV infection, particularly in immunocompromised patients where persistent infection is frequent. Altogether, our work highlights respiratory virus-specific interference in airway epithelia, underlines the key role of IFNs and in particular of IFN-λ in this differential interference and emphases the therapeutic interest of IFN-λ to treat RV infections in at risk populations such as preschool children with recurrent wheezing, asthmatic patients or immunosuppressed hosts.

## Methods

All methods were performed in accordance with the relevant guidelines and regulations.

### In vitro reconstituted airway epithelial tissues

Mucilair is a commercially available *in vitro* reconstituted three-dimensional (3D) airway epithelium. It is produced by Epithelix SàRL (Geneva, Switzerland) according to the Declaration of Helsinki on biomedical research (Hong Kong amendment, 1989), written informed consent was obtained from all subjects involved and the research protocol was approved by the local ethics committee (commission cantonale d’éthique de la recherche CCER de Genève). Culturing of epithelial tissue was performed in an air-liquid interface system (ALI) system at 37 °C and a 5% CO_2_ atmosphere, as previously described^20,38–40^.

### Viruses and viral stock preparation and titration

The following four clinical strains were tested in this study: RV-A16, H1N1, RSV-A, and OC43. The clinical specimen used to prepare the viral stocks were previously screened with a commercially available multiplex real-time RT-PCR kit (Fast-Track Diagnostics; # FTD-2) aimed to detect 21 respiratory pathogens (Flu A, B, H1N1, HCoV NL63, 229E, OC43, HKU, parainfluenza 1, 2, 3, 4; human metapneumovirus A/B; RV, RSV A/B; enterovirus, parechovirus, bocavirus, adenovirus, mycoplasma pneumoniae and internal control). Only samples positive to one single respiratory virus were selected. After ethical approval from the CCER, infected respiratory specimens collected from anonymous patients were inoculated directly in MucilAir without any amplification in the cell lines to avoid adaptation. To prepare viral stocks, five tissues were inoculated in parallel for each virus and apical washes collected from the different epithelia were pooled as previously described^20,38–40^. Viral stocks were then clarified by centrifugation (10 min at 1,500 rpm and 4 °C), aliquoted, and stored at −80 °C. For viral titration, tissues were inoculated in duplicate with serial dilutions of each viral stock. The endpoint dilution was determined based on the presence or absence of viral RNA in the apical wash five days post-infection. In each experiment, the viral inoculum was normalized accordingly to contain the same number of infectious viruses.

### Single and multiple infections in epithelial tissues

MucilAir tissues were treated with 250 µl of a phosphate-buffered saline (PBS) solution containing Ca^++^Mg^++^ (Gibco 14040091; Thermo Fisher Scientific, Waltham, MA, USA) for 45 min at 37 °C under a 5% CO_2_ atmosphere, prior to inoculation with 100 µl of diluted virus. After four hours of adsorption at 33 °C and 5% CO_2_, unbound viral particles were removed and tissues were washed three times using PBS Ca^++^Mg^++^ before incubation at 33 °C and 5% CO_2_ at the ALI. Every 24 hours thereafter, apical washes were collected by applying 200 µl of MucilAir medium apically for 20 min at 33 °C to analyze the daily apical viral production. The culture medium in the basal chamber was also collected daily and replaced with 500 µl of MucilAir medium. For sequential and coinfections, the same infection protocol was used to infect tissues with a second respiratory virus either two days after the infection with the first virus or at the same time, respectively. To quantify cell-associated viruses, tissues (pre-infected or not by a different virus, RSV or H1N1) were infected with RV for four hours, extensively washed to remove unbound viruses, and lysed to quantify cell-associated RV.

### Tissue pre- and cotreatment assays

For type I and type III IFN treatments, 2000IU/ml of IFN-α2a (Roche, Roferon A, Basel, Switzerland) and 5 ng/ml of recombinant Human IL-29/ IFN-λ1 (1598-IL-025; R&D Systems, Minneapolis, MN, USA) were added every day in the basal medium of the ALI tissue culture either from the beginning of the infection in the case of cotreatment or 24 hours before in the case of pretreatment, respectively. For pretreatment with basal samples, the basolateral media of tissues infected for 48 hours were transferred into the basal compartment of new noninfected tissues. Twenty-four hours later, the pretreated epithelium was infected by RV-A16 and fresh basal IFN-free medium was added. The same assays were also performed in the presence of 10 μg/ml of anti-type III IFN-neutralizing antibody (anti-IFN-λ1; MAB15981-100; R&D Systems, Minneapolis, MN, USA) and a mixture of anti-type I IFN-neutralizing antibodies (39000-1, PBL Assay Science) diluted 1/50 in the basal sample during the pretreatment.

### Virus RNA load quantification

Viral RNA was extracted from apical washes using the E.Z.N.A. viral RNA extraction kit (R6874-02; Omega Bio-tek, Inc., Norcross, GA, USA) and from tissue lysate using the E.Z.N.A. total RNA extraction kit (R6834-02; Omega Bio-tek, Inc., Norcross, GA, USA). For RNA quantification, primers and probes specific for each virus were used as previously described^20^. The RNAseP housekeeping gene was used as an internal control for cell-associated virus quantification (4331182; Life Technology, Zug, Switzerland).

Quantitative real-time reverse transcriptase polymerase chain reaction (RT-qPCR) assays were performed using the QuantiTect probe RT-qPCR kit (no. 204443; Qiagen, Valencia, CA, USA) in a StepOne Applied Biosystems thermocycler (Thermo Fisher Scientific, Waltham, MA, USA). In each run and for each virus, four 10-fold serial dilutions of RNA reference standards were included. Results were analyzed using the StepOne version 2.0 software (Thermo Fisher Scientific, Waltham, MA, USA).

### Statistical analysis

Viral load and fold change (FC) in viral load are expressed as means of logarithmic values ± standard errors of the means (SEMs). Statistical significance was determined on logarithmic FC values using one-way or two-way analysis of variance (ANOVA) test (****P < 0.0001, ***P < 0.001, **P < 0.01, *P < 0.05). pValues were calculated with t-test on the area under the curve (AUC) and SD in Fig. 5. All statistics were calculated using the GraphPad Prism version 7.02 software (GraphPad Software, La Jolla, CA, USA).

## Supplementary information

Supplementary Information.

## Data Availability

All data will be available upon request.
