# Author Correction: Interferon-Dependent and Respiratory Virus-Specific Interference in Dual Infections of Airway Epithelia

**DOI:** 10.1038/s41598-020-69600-z

**Published:** 2020-07-22

**Authors:** Manel Essaidi-Laziosi, Johan Geiser, Song Huang, Samuel Constant, Laurent Kaiser, Caroline Tapparel

**Affiliations:** 1 Department of Microbiology and Molecular Medicine, Faculty of Medicine, University of Geneva, Geneva, Switzerland; 2 Division of Infectious Diseases, Geneva University Hospital, Geneva, Switzerland; 3 Epithelix Sàrl, Plan les Ouates, Geneva, Switzerland

Correction to: *Scientific Reports* 10.1038/s41598-020-66748-6, published online 24 June 2020

This Article contains an error in the Results section under the subheading “H1N1 and RSV-A interfere with RV-A16 replication, while RV-A16 does not interfere with either of these viruses” where,

“Co- or sequential infection (with a two-day interval) between RV-A16 and each of the other viruses (using a MOI of around 0.01 viral particles per accessible cell)”

should read:

“Co- or sequential infection (with a two-day interval) between RV-A16 and each of the other viruses (using a MOI of around 0.001 viral particles per accessible cell)”

