# Supplementary material for: Interferon-Dependent and Respiratory Virus-Specific Interference in Dual Infections of Airway Epithelia
Source: Sci Rep. 2020 Jun 24;10:10246. doi: 10.1038/s41598-020-66748-6 (PMC7314816; doi:10.1038/s41598-020-66748-6)
Supplement: Supplementary file 1 — Supplementary Information. [file 41598_2020_66748_MOESM1_ESM.pdf]

INTERFERON-DEPENDENT AND RESPIRATORY VIRUS-SPECIFIC INTERFERENCE IN DUAL INFECTIONS OF AIRWAY EPITHELIA

SHORT TITLE: Viral interference in human respiratory epithelia

Manel Essaidi-Laziosi[1]; Johan Geiser[1]; Song Huang [3]; Samuel Constant[3]; Laurent Kaiser[1,2] and Caroline Tapparel [1,2*]

1. Department of Microbiology and Molecular Medicine, Faculty of Medicine, University of Geneva, Switzerland
2. Division of Infectious diseases, Geneva University Hospital, Switzerland
3. Epithelix Sàrl, Plan les Ouates, Geneva, Switzerland

*Correspondence to: caroline.tapparel@unige.ch

SUPPLEMENTARY METHODS

LACTATE DEHYDROGENASE ASSAY.

LDH release was measured with the Cytotoxicity Detection Kit (Roche 04744926001) according to the manufacturer's instruction. Fifty µl of basal sample was incubated with 50µl of catalyst/dye solution (1/45) for 15' at RT in the dark. The reaction was stopped with 25µl of stop solution. Sample absorbance was measured at 490nm using the photometer reader (Thermo Fisher Scientific Multiskan Go). Percentage of cytotoxicity was calculated with the equation: % of cytoxicity (sample) = 100*[OD(sample)-OD (non-infected control)]/OD(triton treated tissue)-OD(non-infected control)], where triton treated and non-infected tissues constitute the positive and negative controls respectively.

CYTOKINE SECRETION

Interleukin-8 (IL-8 or CXCL-8), CXC motif chemokine 10 (CXCL10 or IP-10) C-C motif chemokine 5 (CCL5 or RANTES), interferon lambda (IFN-λ1/ λ3, IL-29 /IL-28B), and interleukin-6 (IL-6) were measured in the basal medium by ELISA according to the manufacturer's instruction (R&D DY208-05, DY266-05, DY278-05, DY1598B-05, and DY206-05). Sample absorbance was measured at 450nm using the photometer reader (BioTek, FilterMax F5 Multi-Mode Microplate Reader).

SUPPLEMENTARY FIGURES

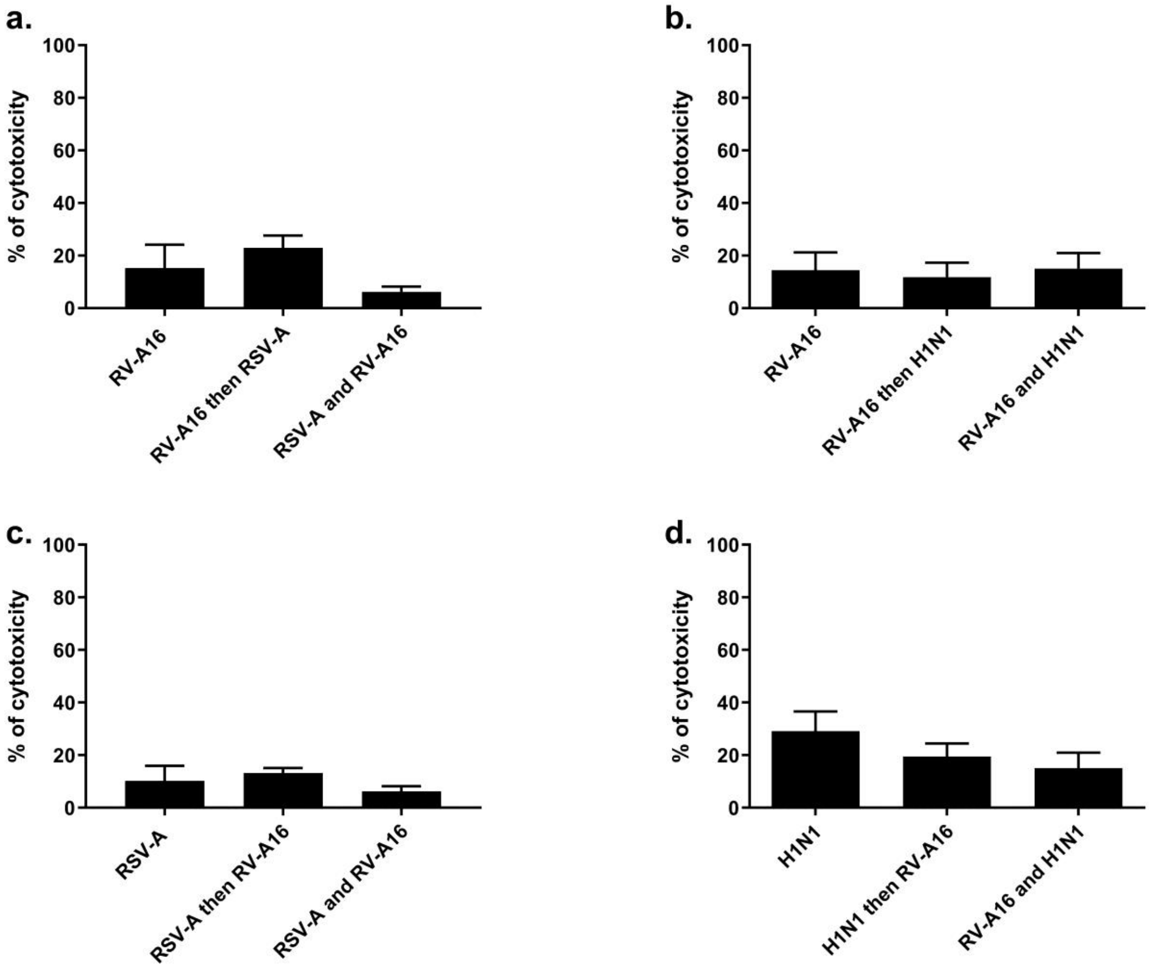

Figure S1: Viral toxicity in dual versus single infections of reconstituted human airway epithelia. Each virus was inoculated alone or in combination with the other virus at the same time or two days after, respectively. Lactase dehydrogenase release was measured as a marker of cell death as previously described (20) at five days after inoculation with the first virus. The percentage of cytotoxic effect was then calculated in comparison with non-infected tissue (0%) and Triton X-100–treated tissue (100%). The analyzed viral couple is specified on the top of each panel, while the chronology of infection is shown on the X-axis (‘then’, after two days; ‘and’, at the same time). Statistical significance relative to single infection was calculated using one-way ANOVA. Data are expressed as mean and SEM of at least three replicates.

The analyzed viral couple is indicated on the top of each panel while the chronology of infection is shown on the X-axis (‘then’, after two days; ‘and’, at the same time). Data represent the means and SEMs of two independent experiments.

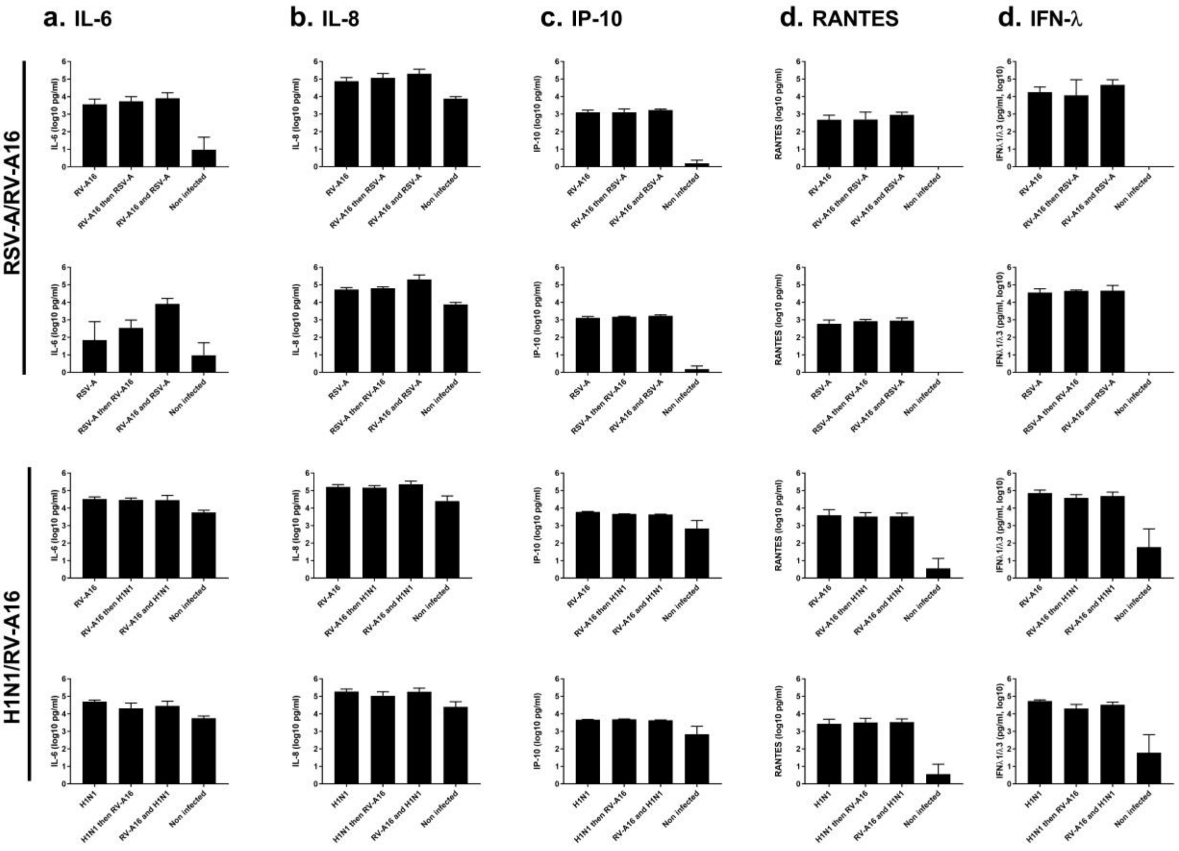

Figure S2: Cytokine induction in dual versus single infections of reconstituted human airway epithelia. Each virus was inoculated alone or in combination with the other virus at the same time or two days after, respectively. IL-6 (a), IL-8 (b), IP-10 (c), RANTES (d) and IFN-λ (e) released in the basal medium were measured by ELISA four DPI with the first virus, as previously described (20). The analyzed viral couple is indicated on the top of each panel while the chronology of infection is shown on the X-axis (‘then’, after two days; ‘and’, at the same time). Data represent the means and SEMs of three replicates.

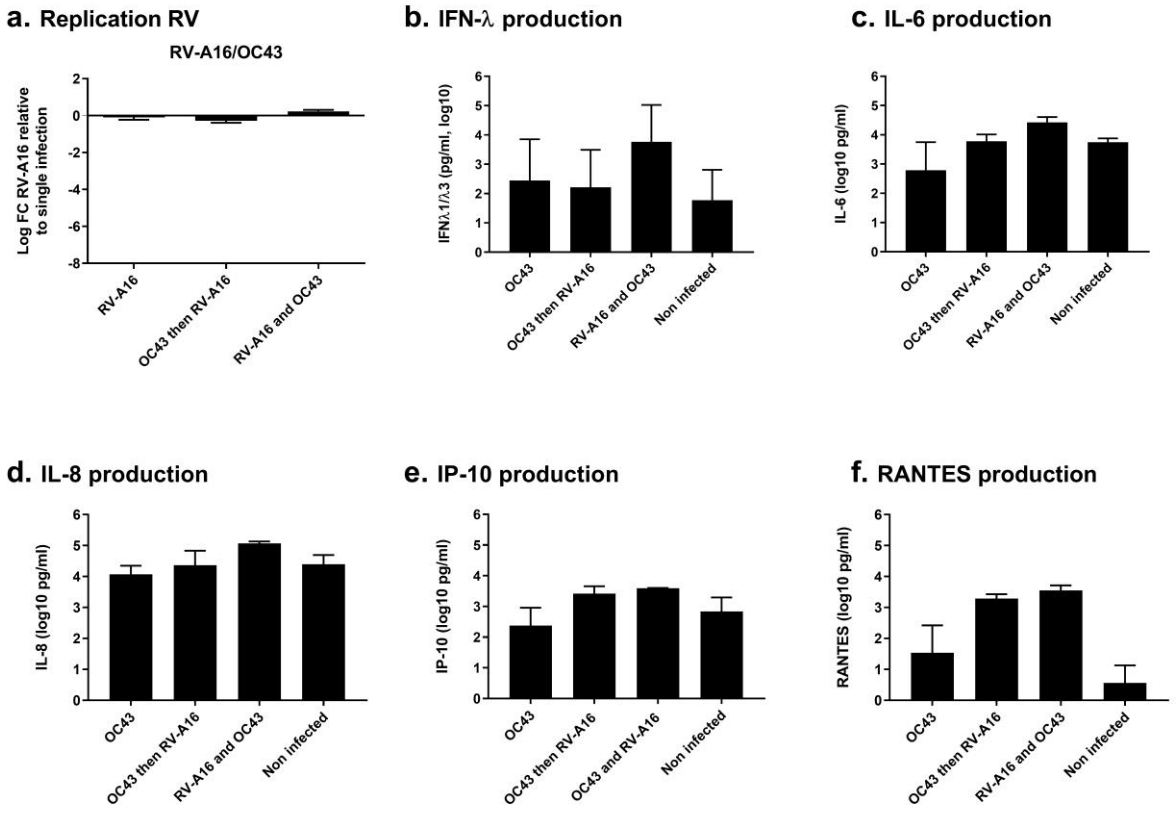

Figure S3: Change in viral replication and cytokine response in dual versus single infections of reconstituted human airway epithelia with OC43 and RV-A16. A. RV-A16 was inoculated alone, at the same time ('and') or two days after (then') OC43. The log fold change (FC) in apically released RV-A16 (measured by RT-qPCR five days post infection) in dual versus single infection is indicated on the Y-axis. The chronology of infection is shown on the X-axis ('then', after two days; 'and', at the same time). **b to f**: IFN-λ (b), IL-6 (c), IL-8 (d), IP-10 (e), and RANTES (f) released in the basal medium four DPI with OC43 were measured by ELISA as previously described [20]. Data are expressed as mean and SEM of at least three replicates.

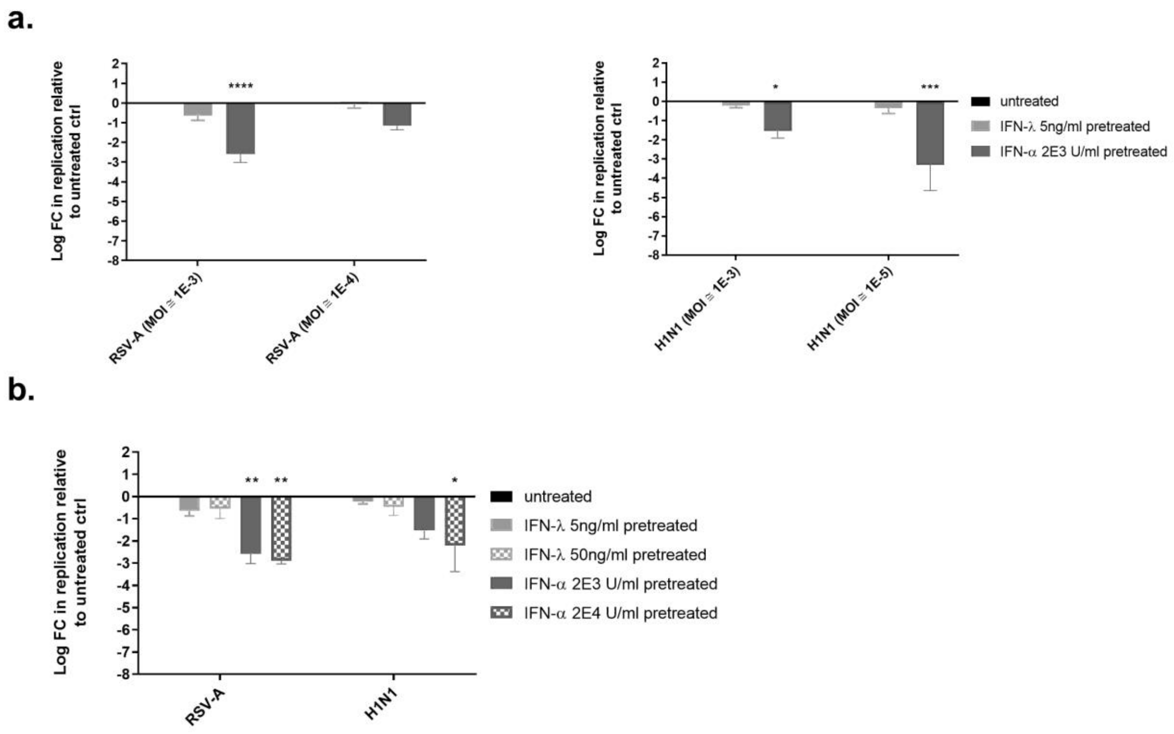

Figure S4: Susceptibility of RSV-A and H1N1 to IFNs after variation of the viral inoculum (a) or the IFN dose (b). Tissues were treated with IFN before and during infection as in figure 4 but with different viral inoculum (MOI around 1E-3 and 1E-4 virus/accessible cell for RSV and 1E-3 or 1E-5 for H1N1) in panel a or different doses of IFN (5ng/ml or 50ng/ml of IFN-λ and 2E3 U/ml or 2E4 U/ml of IFN-α) in panel b. For each condition, the log fold change (FC) in apically released virus (measured by RT-qPCR three days post infection) relative to untreated Ctrl is indicated on the Y-axis. Statistical significance relative to the untreated control was calculated using two-way ANOVA (****P < 0.0001, ***P < 0.001, **P < 0.01, *P < 0.01). Data are expressed as mean and SEM of at least two replicates.
